# Early Orthodontic Treatments of Unilateral Posterior Crossbite: A Systematic Review

**DOI:** 10.3390/jcm10010033

**Published:** 2020-12-24

**Authors:** Francesco Caroccia, Francesco Moscagiuri, Luigi Falconio, Felice Festa, Michele D’Attilio

**Affiliations:** Department of Medical, Oral and Biotechnological Sciences, University of Chieti-Pescara, 66100 Chieti, Italy; fcaroccia20@gmail.com (F.C.); francesco.moscagiuri@unich.it (F.M.); luigifalconio13@gmail.com (L.F.); felice.festa@unich.it (F.F.)

**Keywords:** orthodontics, palatal expansion technique, malocclusion, crossbite, dentofacial orthopedics, craniofacial, systematic review

## Abstract

We aimed to report an update of the systematic review by Petrén et al. (2003). The objective was to evaluate how orthodontic treatments can affect unilateral posterior crossbite (UPXB) in primary and early mixed dentition. Several databases were consulted, and articles published between January 2002 and March 2020 were selected. This review examines the following studies: randomized clinical trials, prospective and retrospective studies with concurrent untreated or normal control groups, and clinical trials comparing at least two treatment strategies. Among the 1581 articles retrieved from the searches, 11 studies were included. Quad-helix (QH) and expansion plate (EP) appliances were compared in three studies. One study compared rapid maxillary expansion (RME) treatment anchored on primary dentition otherwise on permanent molars. One study compared RME and a modified RME with arms extended until deciduous canine and EP. Four studies evaluated the effects of expansion appliances compared with a control group. Compared with the previous review, the quality of the included studies is higher. However, heterogeneity of treatments, different strategies in measurements, lack of a similar follow-up length, and absence of a cost-effectiveness analysis preclude the possibility of providing reliable scientific evidence on the most effective UPXB treatment in primary and early mixed dentition.

## 1. Introduction

### 1.1. Background

Crossbite is a type of malocclusion due to negative transverse discrepancy between maxilla and mandible when the two arches occlude [1]. It can be bilateral or unilateral. Posterior crossbite (PXB) is an abnormal buccal–lingual relationship between premolars and/or molars of the opposing arches in centric occlusion [2]. When the malocclusion affects only one side of the mouth (unilateral posterior crossbite, or UPXB), the lower jaw may have to move to the opposite side to allow molars and premolars to meet with the opposite teeth [3]. This specific type of mandibular shift is known as functional crossbite (FXB) and often leads to a midline deviation. Maxillary expansion is the most common strategy adopted to solve this malocclusion [4].

PXB occurs preferentially in deciduous and mixed dentitions, with prevalence rates ranging from 7.5% to 22% [5,6,7,8,9,10,11]. The most common PXB is the unilateral type, which is usually a functional shift of the jaw toward the crossbite side. Its frequency spans from 80% to 97% of PXB cases [12]. The prevalence of FXB is 8.4% in early dentition, while it decreases to 7.2% in mixed dentition [12]. Suggested factors in crossbite etiology include crowding, premature loss or retention of deciduous teeth, palatal cleft (with or without cleft of the lip), arch deficiencies, and thumb-sucking [12].

Previous studies analyzed the association between UPXB and craniomandibular asymmetry [13], and several authors have suggested that early treatment of UPXB is necessary to avoid long-term effects on normal growth of jaws and teeth [14,15]. Treatment of UPXB induced favorable changes in the kinematics of the mandible [16] and normalized asymmetric functional aberrations as well as stomatognathic muscle activity [11]. Otherwise, failure to treat UPXB caused activity alterations of some chewing muscles (i.e., masseter and temporal muscles) in children and promoted craniomandibular disorders in adolescents [5,17].

FXB is caused by reduced development of maxillary bone starting in the deciduous teeth. This generates a difference between the centric and the maximum intercuspidal position leading to an unstable occlusion and to a consequent shift of the jaw in maximum occlusion. This, in turn, results in a functional unilateral crossbite (FUXB) with midline deviation [18]. If the problem is left unsolved, subsequent craniofacial growth in patients with FUXB may result in facial asymmetry [6,14,19,20]. Facial asymmetry is due to hard and soft tissue adaptation with a consequent increased development of the noncrossbite side and an underdevelopment of the opposite side [21].

The importance of UPXB early treatment has been debated by several systematic reviews [19,22]. However, there is no consensus or clear strategy that could assist in approaching this problem in daily clinical practice [14,22]. Indeed, Petrén et al. [22] in 2003 concluded that it was not possible to obtain scientific evidence showing which treatment modality is the most effective. A similar conclusion was drawn by another review by Agostini et al. [3] published in 2014, in which the authors barely stated low- to moderate-quality evidence to suggest that the quad-helix appliance (QH) may be more successful than removable expansion plates (EPs) at correcting PXB for children in early mixed dentition (aged 8–10 years). However, Agostini et al. [3] did not separate treatment of UPXB or PXB.

Both studies [3,22] in their conclusion called for a future systematic review regarding the same topic including more randomized clinical trials (RCTs) with sufficient sample size, better quality, and homogeneity.

Nowadays, in the era of evidence-based medicine, systematic reviews are of utmost importance in guiding treatment choices.

### 1.2. Objective

This study is an update of a previous review published in 2003 by Petrén et al. [22] and aims to address the following questions:

- Is early treatment of UPXB effective?
- Which treatment modality is the most effective?
- Is the result of treatment stable and long lasting?

## 2. Materials and Methods

### 2.1. Protocol

In undertaking this systematic review, a search and selection strategy was developed by following standards and guidelines reported in the PRISMA Statement [23].

### 2.2. Eligibility Criteria

Details on inclusion and exclusion criteria are reported in Table 1. Data on treatment effects were extracted from the following studies: randomized clinical trials (RCTs), prospective and retrospective studies with normal or untreated control groups, and clinical trials comparing at least two treatment strategies without a control group. Abstracts, case reports, case series, reviews, and opinion articles were excluded.

### 2.3. Information Sources and Search Strategy

**Figure 1:**
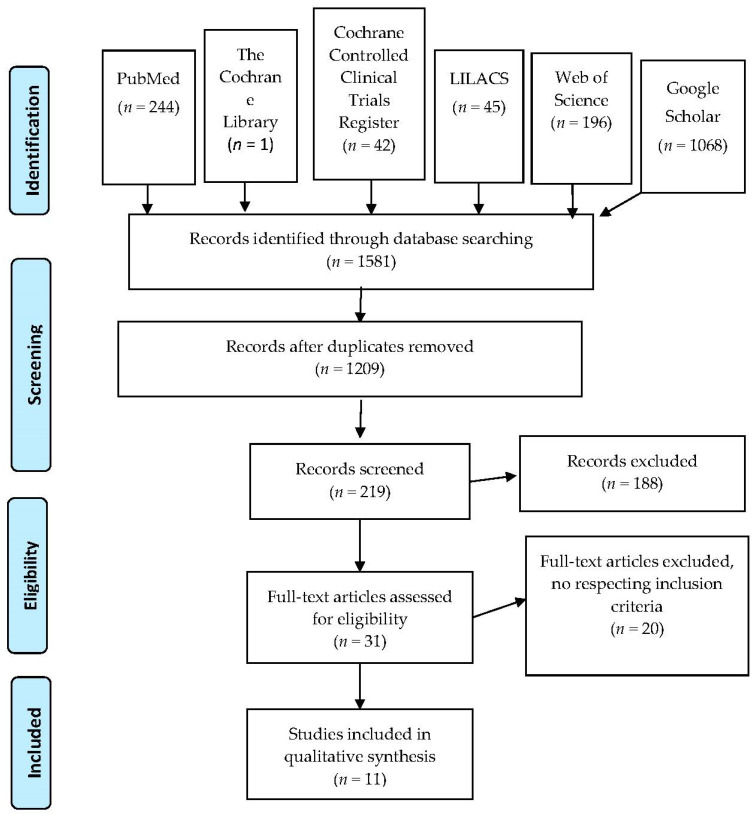
PRISMA flowchart.

The following databases were searched: Medline Database (Entrez PubMed), the Cochrane Library, the Cochrane Controlled Clinical Trials Register, Web of Science, LILACS, and Google Scholar. Reference lists of retrieved articles were hand-searched for additional studies (Figure 1). The survey covered the period from January 2002 to March 2020 and used the MeSH (Medical Subject Headings) terms: “palatal expansion technique” crossed with “Tooth, Deciduous” and “Dentition, Mixed.” Key words were extracted from our questions using the PICOS model (population, intervention, comparison, outcome, studies) (Table 2). The population was composed of children with UPXB in primary or mixed dentition, while controls were individuals with no treatment. Our intervention modality was palatal expansion with different techniques and the outcome of the resolution of the crossbite. No language restrictions were imposed and a search filter for human studies was applied.

### 2.4. Study Selection

Two independent reviewers performed various screens on the initial collection of articles. A first screen was performed by examining titles and abstracts. Subsequently, full texts were assessed for eligibility criteria (Table 1). Disagreements between reviewers were resolved by discussion until a consensus was reached.

### 2.5. Data Items and Data Collection

Retrieved data were collected into Table 3, reporting the following items: year of publication, study design, materials and age, methods/measurements, treatment/retention time, success rate, obtained expansion, remaining expansion, side effects, costs, and author conclusions. Data were extracted by two reviewers without blinding. Once again, disagreements between reviewers were resolved by discussion.

### 2.6. Risk of Bias in Individual Studies

Following the guidance of the Cochrane Handbook [29], the risk of bias in the selected randomized and nonrandomized studies was assessed using the Cochrane’s risk-of-bias tool [29] and the ROBINS-I (Risk of Bias in Non-randomized Studies of Interventions) tool [30], respectively.

### 2.7. Data Synthesis and Summary Measures

To evaluate the expansion effects between pre- and post-treatment, the main summary measure was the difference in means between the intermolar and the intercanine width. Success rate was evaluated by comparing treatment efficiencies, while the most convenient treatment was determined by a cost-effectiveness analysis.

## 3. Results

### 3.1. Study Selection

The search strategy resulted in 1581 articles, and according to title content, 219 articles were selected. After carefully reading the abstracts, 31 articles were retained, and their full texts were checked for compliance with the inclusion/exclusion criteria shown in Table 1. Finally, 11 articles were selected (Figure 1). Of these, only nine studies were subjected to further analysis since, in two cases, articles referred to the same clinical trial. We mostly excluded trials not comparing at least two treatment strategies (case series), studies regarding treatments of permanent dentition/adult patients, and treatments comprising extractions or followed by full-fixed appliances. Case reports, orthognathic surgeries, and studies regarding patients with bilateral crossbite and with cleft lip and/or palate or any other craniofacial syndrome were also excluded.

### 3.2. Study Characteristics

The design of the selected studies is shown in Table 4, and the extracted data are summarized in Table 3. Five RCTs were included.

Out of nine studies, two were performed in Sweden [5,8,26], two in Germany [18,27], one in Italy [24,25], two in Canada [7,10], one in Slovenia [28], and one in Brazil [9].

Three studies compared quad-helix (QH) and expansion plate (EP) treatment modalities [8,9]. One study compared rapid maxillary expansion (RME) treatment anchored on primary dentition with RME anchored on permanent molars [28,29]. One study compared RME and a modified RME (m-RME) with arms extended until deciduous canine and EP [27]. Three studies evaluated the effects of a specific appliance compared with a control group [10,18,28]. In one case, a Haas-type appliance [10], in another case bonded Hyrax (BHY) [18], and in one more case fixed expansion plates (FEPs) were used [28]. Finally, one study assessed a group treated with QH, Hyrax (HY), or Haas-type acrylic coverage modified appliances for rapid maxillary expansion (H-RME) and compared them with a control group [7].

### 3.3. Risk of Bias in Individual Studies

One [9] of five RCTs was judged to have a low risk of bias, one [5,26] had an unclear risk of bias because of unclear randomization of the subjects, and the other three [8,18,24,25] were judged to have a high risk of bias because of the item’s cost-effectiveness analysis or expansion remained, or both were not reported (Supplementary Table S1). Of the four nonrandomized studies, the quality of one [10] was judged to be low risk of bias, two [27,28] as moderate risk of bias, and one [7] as serious risk of bias (Supplementary Table S2).

### 3.4. Results of Individual Studies

#### 3.4.1. Success Rate

The success rate was not explicitly reported in all the studies. QH and RME (although it could be under the form of m-RME or H-RME) appear to achieve expansion of the palate and resolution of the crossbite in 100% of cases (Table 3) [5,8,24,25]. Concerning EPs, the reported success rate was between 66% and 100%, and such a variation is probably due to the lack of collaboration of some patients using a removable appliance [8].

To avoid possible endodontic or periodontal problems on permanent molars, one study randomly compared the expansion associated with H-RME anchored on deciduous with that associated with H-RME anchored on permanent molars [24,25]. In both groups, similar palatal expansion was achieved [24,25]. Furthermore, they showed that anchoring the appliance on deciduous molars (E) leads to more stable expansion in the anterior region of the arch and reduces molar angulation, so that endodontic and periodontal problems on permanent teeth are avoided [24,25].

To correct the crossbite, one study tested the use of composite onlay on mandibular first molar for bite raising [8]. The group with composite onlay was clinically equal to an untreated group. In this case, the bite raising inhibited the forced lateral movement and mandibular locking, but the natural development of the maxilla was not sufficient to self-correct the crossbite [8].

Studies with an untreated control group did not show a spontaneous correction of the crossbite [8,9]. Actually, the increase in maxillary dimension during growth was not sufficient to self-correct the crossbite [8]. This conclusion was drawn from studies in which sucking habits were stopped at least 6 months before the start of the trial. Indeed, in some cases, stopping this habit was shown to lead to a spontaneous correction of the crossbite [8,9].

#### 3.4.2. Treatment Time and Expansion Effects

In all expansion treatments, the period of appliance activation was followed by a period of retention with the same appliance used during the activation period or with another appliance [18]. Less than half of the studies suspended the activation period after reaching overcorrection with the palatal cusp of the maxillary first molar touching the buccal cusp of the first mandibular molar [10,24], or after overexpanding 1 mm on each side [7]. One study supported the notion that overcorrection might be unnecessary, as crossbite correction without overexpansion still showed long-term stability [5].

The average retention time for all the treatments was 6 months. The average treatment length to correct the UPXB was higher with EPs than QH (Table 3).

Not all studies used the same method to evaluate the expansion reached and the expansion remaining. Some used linear measurements such as intercanine or intermolar width, others calculated the surface or the volume of the palate. Linear measurements were not calculated between the same points. Indeed, reference points were the gingival margin, the mesiobuccal cusp tips of first molars, the buccal cusp tips of canines, or the center of occlusal fossa for molars. Table 3 reports the length between the buccal cusps of canines for the item “intercanine width” and the length between the palatal gingival margin of first molars for the item “intermolar width”. One study did not use linear measurements such as intercanine or intermolar width [28]. According to Primožic et al. [28], this method could not exclude bias in assessing treatment success of crossbite correction due to the buccal teeth tipping. Thus, they calculated the increase in surface and volume of the palate with 3D laser scanning technology [28].

The mean expansion obtained immediately after treatment with QH varied between 3.7 and 5.7 mm in the molar region and between 2.0 and 3.5 mm in the canine region. For the EP treatment, the corresponding figures ranged from 3.0 to 4.6 mm in the molar region and from 1.8 to 3.6 mm in the canine region, while those for RME (indeed it was m-RME or H-RME) fell within the range of 4.3–6.4 mm and 2.8–5.1 mm, respectively.

In half of the articles, the expansion effect was followed longitudinally [5,7,9,24,25,27]. However, there was a large difference in follow-up times, ranging from 7 months to 5 years. Thus, for QH, the remaining expansion (i.e., expansion after retention or follow-up) varied from 2.8 to 4.31 mm in the molar region and from 2.9 to 3.2 mm in the canine region. For EP, the remaining expansion varied from 2.6 to 4.4 mm and 1.4 to 2.9 mm in the molar and the canine regions, respectively. Finally, the expansion in the molar and the canine regions for RME appliances fell within the range of 4.0–5.5 mm and 2.4–4.0 mm, respectively (Table 3).

#### 3.4.3. Comparison of Expansion Effects between Treatment Strategies

Studies reported no significant difference in terms of expansion between fixed appliances such as QH [5,8,9] or RME [27] and removable appliances such as EP. One study showed that expansion through an EP with a bite plate cemented on upper primary molars had skeletal effects [28]. According to these authors, PXB correction after active expansion is produced, in part, by the bone apposition in the midpalatal suture and, in part, by the alveolar tipping. While the increase in palatal volume can result from both effects, it is mainly bone apposition in the midpalatal suture that can increase palatal surface area [30]. Studies comparing an H-RME appliance anchored either on deciduous or permanent molars showed that the first option had a successful palatal expansion without endodontic and periodontal problems on permanent teeth [24,25]. Additionally, anchoring the H-RME appliance on deciduous teeth reduced tipping of the permanent molars and achieved a major increase in intercanine width [24,25].

#### 3.4.4. Side Effects and Costs

Two studies [9,26] evaluated costs of treatments and two studies [9,25] their possible side effects (Table 3). Only one study reported the possibility of periodontal and endodontic problems on permanent molars when RME was anchored on them [24,25].

#### 3.4.5. Synthesis of Results

Heterogeneity of treatments modalities, differences in data collection and follow-ups, and absence of a cost-effectiveness analysis in all the studies did not allow us to carry out a synthesis of results by a meta-analysis. Each treatment modality was subjected to a crude evaluation of measurements collected pre- and post-treatment and, whenever it was possible, during follow-up. A cost-effectiveness analysis was carried out by a descriptive evaluation.

## 4. Discussion

This systematic review is an update of a previous review on the same topic published in 2003 by Petrén et al. [22]. Here, similar protocols for database search and data collection were adopted. The literature search covered the period from January 2002 to March 2020. All RCTs or clinical trials, and all prospective and retrospective observational studies with concurrent controls or comparing different treatment modalities for early treatment of UPXB were examined. In summary, it was difficult to draw any solid conclusion from this type of analysis, mostly because of the heterogeneity of treatments and because of differences in data collection and follow-ups. A variety of appliances were compared in these studies and too many different methods were used to take measurements.

### 4.1. Summary of Evidence

In most studies (six out of nine), the sample size was large enough to make reported differences statistically significant [5,8,9,10,18,25]. Dropouts were observed in two out of nine studies [5,18], but their number was, generally, low. The presence or absence of confounding factors, which was correctly stated in most of the studies, is useful information in evaluating the results [5,8,9,10,18,28]. An important etiological factor of UPXB is the sucking habit [12], and this confounding factor was declared and eliminated in four [5,8,9,18] of the nine studies. In all studies, methods used to detect and analyze the effect of treatments were valid and well stated. However, only five studies [7,9,10,18,25] included a method error analysis, and five studies [5,8,9,10,25] declared blinding in measurements or analyses. Compared to the systematic review by Petrén et al. [22], most of the studies in our review present a sufficient sample size, an adequate statistical analysis, and an accurate evaluation of confounding factors. Moreover, half of the studies included an assessment of the long-term stability of UPXB treatments (four out of nine) [5,7,9,24,25,27] after follow-up, but only two [9,25] declared any side effect.

Variability in the materials and methods used in the studies included in this review remained almost the same compared to the previous review. This, as observed by Petrén et al. [22], prevents drawing scientifically valid conclusions regarding the best therapeutic approach to UPXB in deciduous or early mixed dentition. Nevertheless, some important considerations can be drawn.

Petrén et al. [22] reported that grinding has beneficial effects on correction of UPXB in primary dentition. In another review, Harrison and Ashby [19] also concluded that removal of premature contacts in primary teeth is effective in preventing a posterior crossbite from being perpetuated to the mixed dentition and adult teeth. The authors [19] also concluded that when grinding alone is not effective, an upper removable EP could be used. Despite this evidence, none of the articles included in this study evaluated grinding as a possible treatment.

In agreement with two independent reviews [6,22], our results show that QH and RME are reliable appliances for UPXB treatment with about a 100% success rate, which is higher than that achieved by EP.

The presence of trials [8,9] with untreated control groups shows that self-correction of UPXB does not occur, leading to the important clinical implication that correction of UPXB requires orthodontic appliances.

The main measurement technique used in the articles included in this review was linear measurement on models or digital casts. One study [28] proposed a 3D laser scanning technology that could be a new technological method to assess the palatal surface area and palatal volume during maxillary expansion. An alternative technique which was not used in the articles included in this review is cone beam computed tomography (CBCT). CBCT is an effective method in orthodontics diagnosis and an indispensable aid in daily clinical practice [31]. This technique is reliable also to assess transverse maxillary dimensions as demonstrated in a review [32] comparing different RME appliances and expansion protocols.

Only two studies reported a cost-effectiveness analysis of treatments [9,26]. According to Godoy et al. [9], 4% of QH and 27.3% of EP appliances had complications. QH had 33.3% displacement and 18.2% breakage but no cases of appliance loss, while EP had 24.2% loss but no displacement or breakage [9]. Additional laboratory costs due to appliance loss imply that EP is a more expensive treatment [9]. QH treatment is faster, 11% cheaper than EP, and therefore a more cost-effective choice [9]. Additionally, QH had lower direct and indirect costs and fewer failures needing retreatment when compared with EP [23]. Another literature review [6] confirmed that treatment time and cost are higher for EP compared with QH due to poor compliance and lost appliances. There were no studies evaluating costs of the RME appliance; given that this appliance had about a 100% success rate in treating UPXB, it is important to have a cost analysis before claiming that RME is the most appropriate appliance.

However, in this review, compared with what was reported by Petrén et al. [22], the number of RCTs and the quality of the studies are higher, perhaps because two [5,8,26] of the studies included in this review were performed by the same authors of the review we are updating.

For future research, it is necessary to increase the number of RCTs following the same protocol for treatments, data records, and follow-up. It will also be of utmost importance to include a cost-effectiveness analysis to have a better evaluation of the UPXB treatment.

### 4.2. Limitations

Articles included in this systematic review showed quite large heterogeneity regarding defined anatomical landmarks during measurement/treatment protocols, appliances, and follow-up lengths. Some treatment strategies were adopted in only one study, precluding the possibility of drawing reliable and solid conclusions. Not all of the trials declared the absence of various clinical factors such as sucking habits, which could influence the treatment of UPXB. This habit should be stopped before starting the treatment to evaluate the real efficiency of the appliance. In addition, the effectiveness of UPXB early treatment should be evaluated by analyzing changes in the stomatognathic system by an electromyographic study or by assessing mandibular growth and mandibular kinematic alterations following its correction. The findings of this review should be interpreted cautiously, and the importance of early treatment of UPXB should not be neglected.

## 5. Conclusions

The main objectives of this systematic review were to evaluate the effectiveness of early treatment of UPXB, find out the most beneficial treatment modality, and observe the long-term stability of the treatment. The quality of the retrieved studies was overall sufficient for the standards of a systematic review, and half of the studies were RCTs. However, the heterogeneity of the treatments, different strategies in measurements, lack of similar follow-up length, and absence of a cost-effectiveness analysis preclude the possibility of providing a solid and conclusive answer to our initial questions.

- None of the studies with an untreated control group showed a spontaneous correction of UPXB in patients without the sucking habit.
- RME appears to have the same results in expansion rate even if it is modified with arms on deciduous canine or if the Haas type is used. Moreover, RME exhibits similar efficiency when bonded on deciduous or permanent teeth.
- All the appliances used in these studies were successful in correcting UPXB except composite onlay. However, heterogeneity in treatment protocol and different strategies in measurements did not allow us to show which of the treatment modalities (QH, EP, or RME) is the most effective. Further, the substantial variation in follow-up length among the studies resulted in the inability to evaluate the long-term stability of treatments.
- Two RCTs considered QH treatment to be a more cost-effective choice than EP.
- To evaluate the best treatment for UPXB in early and mixed dentition, more RCTs following the same treatment protocol, data records, and follow-up length are required. In future studies, it will be important to also include a cost-effectiveness analysis.

## Figures and Tables

**Table 1:** Inclusion and exclusion criteria.

Inclusion Criteria	Exclusion Criteria
Human studies	Case reports and case series
Primary and early mixed dentition with unilateral posterior crossbite	Review articles and abstracts
Randomized controlled trials, prospective and retrospective observational studies with concurrent untreated as well as normal controls	Treatment in late mixed and permanent dentition, adults
Clinical trials comparing at least two treatment strategies	Treatment combined with extraction or full-fixed appliances
	Surgically assisted treatment
	Cleft lip and/or palate or another craniofacial syndrome diagnosis
	Anterior crossbite, Angle class III

**Table 2:** PICOS model for search strategy.

PICOS Model	Description
**P: patients/population**	Children with primary or mixed dentition who have unilateral posterior crossbite
**I: intervention**	Palatal expansion with different techniques
**C: comparison**	No treatment
**O: outcome**	Resolution of the crossbite
**S: study design**	Randomized controlled trials (RCTs), prospective and retrospective studies with concurrent untreated as well as normal controls, and clinical trials comparing at least two treatment strategies without any untreated or normal group involved

**Table 3:** Data analysis. H-RME indicates patients treated with a two-banded Haas-type appliance for rapid maxillary expansion; UC, untreated control group; RME, rapid maxillary expansion; GrE, RME on second deciduous molars; Gr6, RME on first permanent molars; HY, Hyrax; QH, quad-helix; EP, expansion plates; NC, normal control group; CO, composite onlay on mandibular first molar for bite raising; mRME, modified RME with arms until deciduous canine; BHY, bonded Hyrax according to McNamara; FEP, fixed expansion plate with bite plane; BPE bonded palatal expansion; CR, mandibular banded Crozat/lip bumper; TG, treatment group; UPXB, unilateral posterior crossbite; PXB, posterior crossbite.

Study	Device and Age	Methods/Measurements	Treatment Time/Retention Time	Success Rate	Expansion Obtained Molars/Cusps (mm)	Expansion Remained Molars/Cusps (mm)	Side Effects	Long-Term Stability	Costs	Author Conclusion
Bukhari et al. [10] (2018)	30H-RME~8 years 30UC~8 years	Study digital cast	Contacting upper lingual cusps with lower buccal Retention time: 6 months	Not declared	H-RME: 4.8/4.6 UC: 0.5/0.6	Not declared	Not declared	No long-term analysis	Not declared	H-RME expansion greater than UC. Major inclination of molars in H-RME on the cross side.
Ugolini et al. [24] (2015) and Cerruto et al. [25] (2017)	35GrE~8.4 years 35Gr6~8.6 years	Study scanned cast Cephalometric and panoramic radiographs	Contacting upper lingual cusps with lower buccal GrE: 41 days Gr6: 35 days	35/35 35/35	GrE: 4.3/4.2 Gr6: 5.8/3.3	GrE: 4.0/3.8 Gr6: 4.2/3.1 10mo	Periodontal and endodontic problems in permanent molars if banded.	Similar expansion after 10mo of retention. No long-term analysis	Not declared	Similar expansion in GrE and Gr6. GrE avoids endodontic and periodontal problems on permanent molar.
Wong et al. [7] (2011)	TG: 56H-RME~7.7 years 26HY~7.7 years 28QH~7.7 years CG	Study casts Digital caliper	Overexpanded 1 mm each side Retention time: 3 months	Not declared	TG: 4.3/4.6 CG: 0.7/0.6	TG: 3.6/4.5 CG: 2.4/2.5 5 years	Not declared	All appliances produced similar maxillary arch expansion short and long term. 80% intermolar and 98% intercanine stability	Not declared	UPXB patients had narrower maxillary widths than controlsprior to expansion. Post expansion, maxillary intercanineand intermolar widths were significantly greater than controls.
Petrén et al. [5,26] (2011), (2013)	20QH~ 9 years 15EP~8.5 years 20NC~8.8 years	Study casts Digital caliper	QH and EP: until normal transverse relationship (no overcorrection) Retention time: 6 months	19/20 15/15	QH: 3.7/2.7 EP: 3.2/2.6	QH: 2.8/3.2 EP: 2.6/2.5 NC: 2.0/1.6 3y	Not declared	QH and EP have the same long-term stability after 3 years	QH offers significant economic benefits over	QH and EP achieve similar results with a favorable long-term stability.
Petrén and Brondemark [8] (2008)	15QH~9.1 years 15EP~8.7 years 15CO~8.3 years 15UC~8.8 years	Study casts	QH and EP: until normal transverse relationship (no overcorrection) QH: 4.8 months EP: 9.6 months Retention time: 6mo	15/15 10/15 2/15 0/15	QH: 4.4/2.0 EP: 3.0/2.7 CO: 0.3/0.6 UC: 0.3/0.3	Not declared	Not declared	No long-term evaluation	Not declared	QH effective to correct UPXB in early mixed dentition; EP successful in 2/3 of patients due to insufficient collaboration of the others. Composite onlay was not effective. Spontaneous correction in the mixed dentition did not occur.
Weyrich et al. [27] (2010)	20mRME~8.7 years 10RME~9.4 years 10EP~9.5 years	Study casts Electronic Caliper	mRME and RME: ~16.5 days Retention time: 6–7 months EP: 4 monthsRetention time: 13 months	20/20 10/10 10/10	mRME: 5.7/5.1 RME: 6.4/2.8 EP: 4.6/3.6	mRME: 5.0/4.0 RME: 5.5/2.4 EP: 4.4/2.9 7–13 months	Not declared	mRME, RME, and EP have the same stability after retention, but no long-term data are declared.	Not declared	mRME revealed similar effects to RME. EP requires more time but similar results of mRME and RME.
Lippold et al. [18] (2013)	31BHY~7 years 35UC~7 years	CBCT scanning of plaster casts	3.2 weeks Retention time: 4 months + U-bow activator for 9 months	Not declared	BHY: 5.1/3.6 UC: 0.8/1.0	Not declared	Not declared	No long-term evaluation	Not declared	BHY device followed by U-Bow in late deciduous and early mixed dentition is effective.
Primozic et al. [28] (2011)	20FEP~5.2 years 20UC~5.7 years 20NC~5.4 years	3D laser scanning technology	FEP: 4 weeks, reaching a slight hypercorrection Retention time: ~5 months without fixed bite plate	17/20	SURFACE FEP: 75.1 mm^2^ UC: 36.6 mm^2^ NC: 38.1 mm^2^ VOLUME FEP: 389.0 mm^3^ UC: 59.0 mm^3^ NC: 201.8 mm^3^	Not declared	Not declared	No long-term evaluation	Not declared	Both palatal surface and volume in TG statistically significantly increased after treatment. EP in primary dentition could partly have a skeletal effect.
Godoy et al. [9] (2011)	33QH~8 years 33EP~7.8 years 33UC~8.1 years	Study dental castsSliding caliper	Until cross correction (no overcorrection) QH: ~3 months EP: ~4–6 months Retention time: 6mo	QH:33/33 EP:30/33 UC:0/33	QH: 5.7/3.5 EP: 4.4/1.8 UC: 0.1/−0.2	QH: 4.3/3.0 EP: 3.1/1.4 UC: 0.8/0.4 12 months after PXB correction	8EP loss. QH: 11 displaced, 6 broken.	QH major stability than EP. 9.1% relapse after 1year	EP costs 10.53% more than QH.	QH and EP equally effective. QH is more cost-effective choice than EP. UPXB did not spontaneously correct during the transition into permanent dentition.

**Table 4:** Study design of selected articles.

Articles	Study Design
Bukhari et al. [10] (2018)	R, CCT, UC
Ugolini et al. [24] (2015) and Cerruto et al. [25] (2017)	P, RCT
Wong et al. [7] (2011)	R, CCT, NC
Petrén et al. [5,26] (2011), (2013)	P, RCT, NC
Petrén and Brondemark [8] (2008)	P, RCT, UC
Weyrich et al. [27] (2010)	P, CT
Lippold at al. [18] (2013)	P, RCT, UC
Primozic et al. [28] (2011)	P, CCT, UC, NC
Godoy at al. [9] (2011)	P, RCT, UC

P, prospective study; R, retrospective study; L, longitudinal study; RCT, randomized clinical trial; CCT, controlled clinical trial; CT, clinical trial, i.e., comparison at least two treatment modalities without any untreated or normal group involved; UC, untreated control group; and NC, normal control group.

## Data Availability

Data is contained within the article or supplementary material.

## Supplementary Materials

The following are available online at https://www.mdpi.com/2077-0383/10/1/33/s1, Table S1: Summary risk of bias assessment of randomized clinical trial according the Cochrane collaboration’s risk of bias assessment tool, Table S2: Summary risk of bias assessment of the nonrandomized studies according to the ROBINS-I tool.
