# Supplementary material for: Early Orthodontic Treatments of Unilateral Posterior Crossbite: A Systematic Review
Source: J Clin Med. 2020 Dec 24;10(1):33. doi: 10.3390/jcm10010033 (PMC7795353; doi:10.3390/jcm10010033)
Supplement: Supplementary file 1 [file jcm-10-00033-s001.pdf]

Supplementary Table 1. Summary risk of bias assessment of Randomized Clinical trial according the Cochrane collaboration's risk of bias assessment tool

Trial (Author and year)	Random sequence generation (selection bias)	Allocation concealment (selection bias)	Blinding of outcome assessment (detection bias)	Incomplete outcome data (attrition bias)	Selective reporting (reporting bias)	Other sources of bias	Overall Risk of bias
Ugolini et al. [28] and Cerruto et al. [29]	**Low risk** Using a stratified blocked randomization	**Unclear**	**Low risk** Blinding in measurements	**Low risk** No dropouts or patient losses are reported	**High risk** Cost-effective analysis is not reported	**Low risk** No	**High risk**
Petrén et al. [5,26]	**Unclear**	**Unclear**	**Low risk** Blinding in measurements	**Low risk** 5 dropouts are reported but sample size remained sufficient	**Low risk** All outcomes that are of interest in the review have been reported	**Low risk** No	**Unclear**
Petrén and Brondemark [8]	**Low risk** Patients were randomly allocated	**Low risk** Sequentially numbered, opaque, sealed envelopes	**Low risk** Blinding in measurements	**Low risk** No dropouts or patient losses are reported	**High risk** Cost-effective analysis and expansion remained are not reported	**Low risk** No	**High risk**
Lippold et al. [18]	**Low risk** Using a stratified blocked randomization	**Unclear**	**Unclear** No information provided	**Low risk** 11 dropouts are reported but sample size remained sufficient	**High risk** Cost-effective analysis and expansion remained are not reported	**Low risk** No	**High risk**
Godoy et al. [9]	**Low risk**	**Low risk** Numbers randomly drawn	**Low risk** Blinding in measurements	**Low risk**	**Low risk**	**Low risk** No	**Low risk**

	Patients were randomly allocated	from a plastic bag		No dropouts or patient losses are reported	All interesting outcomes have been reported		

Supplementary Table 2. Summary risk of bias assessment of the non-randomized studies according to the ROBINS-I tool

**Risk of bias assessment**					
	**Response**	Bukhari et al. [10]	Wong et al. [7]	Weyrich et al. [27]	Primozic et al. [30]
Bias due to confounding		Low	Low	Low	Low
Bias in selection of participants into the study		Low	Low	Low	Low
Bias in classification of interventions		Low	Serious	Low	Low
Bias due to deviations from intended interventions		Low	Low	Low	Low
Bias due to missing data		Low	Low	Low	Low
Bias in measurement of outcomes		Low	Moderate	Moderate	Moderate
Bias in selection of reported result		Low	Low	Low	Moderate
Overall bias		Low	Serious	Moderate	Moderate
	Low				
	Moderate				
	Serious				

	Critical				
	No information				

Complete Search Strategy

PUBMED

"palatal expansion technique"[MeSH Terms] AND ("dentition, mixed"[MeSH Terms] OR "tooth, deciduous"[MeSH Terms])

THE COCHRANE LIBRARY AND TRIALS

Title, Abstract, Keyword: palatal expansion AND (mixed dentition OR deciduous teeth)

LILACS

(((((palatal expansion) or "PALATAL EXPANSION TECHNIC") or "PALATAL EXPANSION TECHNICS") or "PALATAL EXPANSION TECHNIC/AE") or "EXPANSION MAXILAR/AE") or "MAXILLARY EXPANSION/AE" [Palabras] and ((tooth deciduous) or "DECIDUOUS DENTITION") or "MIXED DENTITION" [Palabras]

WEB OF SCIENCE

(TS = (palatal expansion)) AND (TS = “tooth deciduous” OR “teeth deciduous” OR “mixed dentition”)

GOOGLE SCHOLAR

unilateral posterior crossbite correction in mixed dentition or deciduous
